# Validierung innerklinischer Sichtungsalgorithmen für den Massenanfall von Verletzten – eine simulationsbasierte Studie – deutsche Version

**DOI:** 10.1007/s00101-023-01291-3

**Published:** 2023-06-15

**Authors:** Axel R. Heller, Tobias Neidel, Patrick J. Klotz, André Solarek, Barbara Kowalzik, Kathleen Juncken, Christan Kleber

**Affiliations:** 1 Klinik für Anästhesiologie und Operative Intensivmedizin, Medizinische Fakultät, Universität Augsburg, Stenglinstr. 2, 86156 Augsburg, Deutschland; 2 Interdisziplinäre Notaufnahme, Medizinische Fakultät, Universitätsklinikum Schleswig-Holstein Campus Kiel, Kiel, Deutschland; 3 Stabsstelle Katastrophenschutz, Charité, Berlin, Deutschland; 4 Referat III.3 Schutz der Gesundheit, Bundesamt für Bevölkerungsschutz und Katastrophenhilfe, Bonn, Deutschland; 5 Medizinisches Direktorium, Städtisches Klinikum Dresden, Dresden, Deutschland; 6 Klinik und Poliklinik für Orthopädie, Unfallchirurgie und Plastische Chirurgie (OUP), Universitätsklinikum Leipzig AöR, Leipzig, Deutschland

**Keywords:** Katastrophenmedizin, Notfallvorsorge, Innerklinische Sichtung, Krankenhaus Alarm- und Einsatzplan, Notaufnahme, Disaster management, Emergency preparedness, Secondary triage, Hospital alarm and operation plan, Emergency department

## Abstract

**Hintergrund:**

Die situationsbedingte Verknappung medizinischer Ressourcen endet bei einem Massenanfall von Verletzen (MANV) lageabhängig nicht mit dem Abtransport der Patienten von der Einsatzstelle. Folglich ist in den aufnehmenden Kliniken eine Eingangssichtung erforderlich. Ziel dieser Studie war es im ersten Schritt einen Referenz‐Patientenvignettensatz mit definierten Sichtungskategorien zu erstellen. Dies erlaubte im zweiten Schritt, die rechnergestützte Evaluation der diagnostischen Güte klinischer Sichtungsalgorithmen für MANV-Lagen.

**Methodik:**

In einen mehrstufigen Bewertungsprozess durch zunächst sechs, später 36 Sichtungsexperten gingen 250 in der Übungspraxis validierte Fallvignetten ein. Diese Algorithmen – unabhängige Expertenbewertung aller Vignetten – dienten als Goldstandard für die Analyse der diagnostischen Güte der folgenden innerklinischen Algorithmen: Manchester Triage System (MTS Modul MANV), Emergency severity Index (ESI), Berliner Sichtungsalgorithmus (BER), die prähospitalen Algorithmen PRIOR und mSTaRT, sowie zwei Projektalgorithmen aus einer Kooperation des Bundesamts für Bevölkerungsschutz und Katastrophenhilfe (BBK) mit dem Haschemitischen Königreich Jordanien – innerklinischer jordanisch-deutscher Projektalgorithmus (JorD) und prähospitaler Sichtungsalgorithmus (PETRA). Jede Patientenvignette durchlief computergestützt eine Sichtung durch alle angegeben Algorithmen, um vergleichend die Testgüte für alle Verfahren zu erheben.

**Ergebnisse:**

Von den ursprünglich 250 Vignetten konnte eine Sichtungsreferenzdatenbank mit 210 Patientenvignetten algorithmenunabhängig validiert werden. Diese bildeten den Goldstandard für den Vergleich der analysierten Sichtungsalgorithmen. Die Sensitivitäten für die innerklinische Detektion von Patienten der Sichtungskategorie I lagen zwischen 1,0 (BER, JorD, PRIOR) und 0,57 (MANV-Modul MTS). Die Spezifitäten lagen zwischen 0,99 (MTS und PETRA) und 0,67 (PRIOR). Gemessen am Youden-Index ergab sich bei BER (0,89) und JorD (0,88) die beste Gesamtperformance für die Detektion von Patienten der Sichtungskategorie I. Eine Übertriage ist am ehesten bei PRIOR, eine Untertriage beim MANV-Modul von MTS zu erwarten. Bis zum Entscheid SK I benötigen die Algorithmen folgende Schrittanzahlen (Median [IQR]): ESI 1 [1–2]; JorD 1 [1–4]; PRIOR 3 [2–4]; BER 3 [2–6]; mSTaRT 3 [3–5]; MTS 4 [4–5]; PETRA 6 [6–8]. Für die SK II und III besteht ein positiver Zusammenhang zwischen der Schrittanzahl bis zum Entscheid und der Testgüte.

**Schlussfolgerung:**

In der vorliegenden Studie konnte eine Übertragbarkeit prähospitaler algorithmenbasierter Vorsichtungsergebnisse auf die Ergebnisse klinischer Algorithmen gezeigt werden. Die höchste diagnostische Güte für die innerklinischen Sichtung lieferten BER und JorD, die allerdings auch die meisten Algorithmusschritte bis zum Entscheid benötigen.

**Zusatzmaterial online:**

Die Online-Version dieses Beitrags (10.1007/s00101-023-01291-3) enthält die Sammlung der zusätzlich im Text erwähnten Abbildungen S1 und S2 und die Tabellen S1–S8.

## Abstract (en)

**Background:**

In the event of a mass casualty incident (MCI), the situation-related shortage of medical resources does not end when the patients are transported from the scene of the incident. Consequently, an initial triage is required in the receiving hospitals. In the first step, the aim of this study was to create a reference patient vignette set with defined triage categories. This allowed a computer-aided evaluation of the diagnostic quality of triage algorithms for MCI situations in the second step.

**Methods:**

A total of 250 case vignettes validated in practice were entered into a multistage evaluation process by initially 6 and later 36 triage experts. This algorithm—independent expert evaluation of all vignettes—served as the gold standard for analyzing the diagnostic quality of the following triage algorithms: Manchester triage system (MTS module MCI), emergency severity index (ESI), Berlin triage algorithm (BER), the prehospital algorithms PRIOR and mSTaRT, and two project algorithms from a cooperation between the Federal Office of Civil Protection and Disaster Assistance (BBK) and the Hashemite Kingdom of Jordan—intrahospital Jordanian-German project algorithm (JorD) and prehospital triage algorithm (PETRA). Each patient vignette underwent computerized triage through all specified algorithms to obtain comparative test quality outcomes.

**Results:**

Of the original 250 vignettes, a triage reference database of 210 patient vignettes was validated independently of the algorithms. These formed the gold standard for comparison of the triage algorithms analyzed. Sensitivities for intrahospital detection of patients in triage category T1 ranged from 1.0 (BER, JorD, PRIOR) to 0.57 (MCI module MTS). Specificities ranged from 0.99 (MTS and PETRA) to 0.67 (PRIOR). Considering Youden’s index, BER (0.89) and JorD (0.88) had the best overall performance for detecting patients in triage category T1. Overtriage was most likely with PRIOR, and undertriage with the MCI module of MTS. Up to a decision for category T1, the algorithms require the following numbers of steps given as the median and interquartile range (IQR): ESI 1 (1–2), JorD 1 (1–4), PRIOR 3 (2–4), BER 3 (2–6), mSTaRT 3 (3–5), MTS 4 (4–5) and PETRA 6 (6–8). For the T2 and T3 categories the number of steps until a decision and the test quality of the algorithms are positively interrelated.

**Conclusion:**

In the present study, transferability of preclinical algorithm-based primary triage results to clinical algorithm-based secondary triage results was demonstrated. The highest diagnostic quality for secondary triage was provided by the Berlin triage algorithm, followed by the Jordanian-German project algorithm for hospitals, which, however, also require the most algorithm steps until a decision.

## Einleitung

Das zeitgleiche spontane Auftreten einer größeren Zahl Verletzter oder Erkrankter stellt sowohl präklinische als auch klinische Strukturen in der Notfallmedizin regelmäßig vor größere Herausforderungen [1–3]. Massenanfälle von Verletzten (MANV) sind zumindest anfänglich von einem erheblichen Ressourcenmangel gekennzeichnet [4, 5]. Zu diesem Zeitpunkt im Einsatzverlauf muss es das Ziel sein, die vorhandenen, aber spärlichen Ressourcen so effizient einzusetzen, dass trotzdem das Überleben möglichst vieler Patienten mit nachfolgend bestmöglicher Lebensqualität gesichert werden kann [5–7]. Hierzu dient die frühzeitige abgestufte Identifizierung der Patienten nach ihrem unmittelbaren Behandlungsbedarf, mit entsprechender Klassifikation in eine von 4 Sichtungskategorien (SK I–IV bzw. rot, gelb, grün und blau) und ihre Kennzeichnung [7–9].

Die hier dargestellte Sichtung findet sich in der deutschen juristischen Terminologie als „Ex-ante-Triage“: Sie wird angewendet *„… wenn die Zahl der zu Behandelnden die verfügbaren Mittel überschreitet, so dass zwar vielleicht alle Patienten alternativ Chancen auf eine erfolgreiche Behandlung haben, dies aber nicht gleichzeitig geschehen kann“* [10]. Die deutsche Rechtsprechung sieht dieses Szenario für die Akteure vor Ort als Kollision mehrerer gleichwertiger Handlungspflichten zur Rettung von Leben. Diese Kollision führt *„… nach der gesetzlich zwar nicht geregelten, aber als Gewohnheitsrecht anerkannten Rechtsfigur der rechtfertigenden (Handlungs-/) Pflichtenkollision dazu, dass nicht rechtswidrig handelt, wer nur so viele Menschen wie nach Ressourcenlage möglich rettet“* [10, 11].

Um diese Behandlungspriorisierung binnen kurzer Zeit einheitlich und präzise realisieren zu können, existieren unterschiedliche Vorsichtungsalgorithmen für die Präklinik [12, 13]. Diese wurden in den letzten Jahren zunehmend in Studien evaluiert [6, 13–17]. Die 7. Sichtungskonsensuskonferenz beim Bundesamt für Bevölkerungsschutz und Katastrophenhilfe (BBK) hat 2017 auch auf der Basis vorangegangener Evaluationsdaten unserer Arbeitsgruppe [6] unter Mitarbeit von 22 Fachverbänden u. a. ein Anforderungsprofil für (Vor‑)Sichtungsverfahren erstellt [18].

Die situationsbedingte Verknappung von Ressourcen endet lageabhängig nicht mit dem Abtransport der Patienten von der Einsatzstelle. Vielmehr setzt sie sich in Abhängigkeit von der Nutzung von Patientenverteilungsverfahren [5] in die aufnehmenden Kliniken fort [1, 19]. Hier muss eine erneute Sichtung der Patienten erfolgen; erstens um der Dynamik des Patientenzustands im Zeitverlauf gerecht zu werden [8, 9]. Zweitens muss lagebedingt auch damit gerechnet werden, dass am Einsatzort eine ärztliche Sichtung nicht durchgeführt werden kann [20] und eine erste Behandlungs- und Transportpriorisierung bis zum Krankenhaus nur durch nichtärztliches Personal erfolgt [21].

Um den ersten Arztkontakt der Patienten im Alltag einer Krankenhausnotaufnahme entsprechend ihrer aktuellen Behandlungsdringlichkeit zu priorisieren [3], werden in deutschen Notaufnahmen insbesondere das Manchester Triage System (MTS) [22] und der Emergency Severity Index (ESI) als Ersteinschätzungssysteme eingesetzt [23]. Für ihre Tauglichkeit in MANV-Lagen sind diese beiden Systeme jedoch bisher nicht evaluiert. Spezifisch auf die klinische Sichtung bei MANV zugeschnittene Algorithmen existieren kaum. Der Nutzen derartiger Algorithmen ist für die Präklinik gezeigt [13]. Über- oder Untertriagierungen führen aber dazu, dass Patienten nicht diejenigen Behandlungsressourcen erhalten, die ihrer tatsächlichen Behandlungsdringlichkeit entsprechen [9, 24, 25]. Aktuell sind das MANV-Modul des MTS [22] und der Berliner Sichtungsalgorithmus [24] die einzigen in Deutschland angewendeten Verfahren innerhalb von Krankenhäusern. Der Berliner Sichtungsalgorithmus wurde als einziger bis dato intern und extern validiert [24]. Ein systematischer Vergleich der unterschiedlichen innerklinischen Sichtungsalgorithmen anhand standardisierter Verletzter fehlt.

Ziel dieser Studie war es daher, zunächst analog zu eigenen Vorarbeiten aus der Präklinik [6] einen konsentierten Masterdatensatz an Patientenvignetten mit definierten Sichtungskategorien zu erstellen, die auch die erweiterten diagnostischen Fähigkeiten einer Krankenhausnotaufnahme abbilden. Hiermit ist es erstmals möglich, die vorhandenen klinischen Algorithmen zur Sichtung von Patienten in MANV-Lagen zu evaluieren. Um die Vergleichbarkeit mit Erkenntnissen aus der Präklinik herzustellen, wurden ebenso die weit verbreiteten Vorsichtungsalgorithmen PRIOR [26] und mSTaRT [16] mit in die Analyse aufgenommen. Ebenso werden 2 Projektalgorithmen aus einem internationalen Zivilschutzprojekt des BBK und dem Haschemitischen Königreich Jordanien evaluiert [27, 28].

## Methodik

Aus den Notfallübungen Berliner Krankenhäuser, Dresdner Übungen und Einsatzdarstellungen der Berufsfeuerwehr Berlin standen 250 in der Übungspraxis validierte Fallvignetten ohne Patientenbezug mit entsprechenden medizinischen Angaben zur Verfügung [25]. Entwickelt wurden die standardisierten Vignetten zur reproduzierbaren Vorbereitung der Mimen und als Rollenskripte für die in Berlin regelmäßig stattfindenden Krankenhausnotfallübungen der Jahre 2011–2015 [25]. Die Vignetten enthielten alle relevanten klinischen Informationen, um eine Eingangssichtung im Krankenhaus und Weiterversorgung zu simulieren. Bei entsprechenden Verletzungsmustern standen auch stichpunktartig die Ergebnisse ergänzender Bildgebung (Röntgen, Sonographie, Computertomographie) zur Verfügung. Als Rahmenszenario diente eine außerklinische Großschadenslage, ohne Ressourcenlimitierung des Krankenhauses [25]. In einem zweistufigen Delphi-Verfahren wurden die 250 Fallvignetten durch 6 Mitglieder der 8. Sichtungskonsenskonferenz [8] zunächst unabhängig voneinander und verblindet auf Plausibilität und Vollständigkeit überprüft und einer entsprechenden Sichtungskategorie zugeordnet. In einer zweiten Validierungsrunde wurden uneinheitlich bewertete Vignetten diskutiert. Entweder konnte ein Konsens erzielt und die Fallvignetten entsprechend angepasst werden, oder die Fallvignetten wurden aus dem Vignettensatz entfernt. Ebenso wurden Duplikate aus der weiteren Betrachtung herausgenommen. Am Ende des Vorbereitungsprozesses verblieben 210 vorkonsentierte Fallvignetten zur Fortentwicklung zu einem Referenzdatensatz in der anschließenden breiteren Validierungsrunde durch die 36 Fachexperten.

Zur Bewertungskonsentierung der Sichtungskategorisierung des vorbereiteten Patientenvignettensatzes und zur Fortentwickelung zum Referenzdatensatz wurden die 210 verbliebenen Fallvignetten analog zu prähospitalen Vorarbeiten [6] einer Gruppe von 36 sichtungserfahrenen Notfallmediziner:innen vorgelegt. Die Sichtenden wurden durch das BBK bzw. in Abstimmung mit dem BBK persönlich eingeladen. Die Bewertungskonsentierung hinsichtlich der jeweiligen Sichtungskategorie erfolgte passwortgeschützt auf der Online-Befragungsplattform SoSci Survey [29]. Fünf eindeutige Bewertungsbeispiele wurden den Sichtenden pro Sichtungskategorie zur Orientierung vorgegeben. Sie wurden explizit gebeten, keine Algorithmen anzuwenden, sondern nach ihrer klinischen Erfahrung zu entscheiden. Somit liegen der Bildung der Referenzkategorien 7560 Sichtungsvorgänge zugrunde. Entsprechend der 8. Sichtungskonsensuskonferenz von 2019/2020 [8] konnten für die Eingangssichtung im Krankenhaus nur die Sichtungskategorien SK I (rot), SK II (gelb) und SK III (grün) vergeben werden. Die Sichtungskategorie SK IV (blau) stand entsprechend dem Konsens der beteiligten Fachgesellschaften für die Eingangssichtung im Krankenhaus nicht zur Auswahl. Die Vergabe der Sichtungskategorie SK IV (blau) kann im Rahmen einer Reevaluation im Behandlungsbereich der SK I (rot) bei tatsächlichem Ressourcenmangel erfolgen [8]. Als vignettenindividueller Referenzwert der Sichtungskategorie wurde der Median der von den 36 Sichtenden festgelegten Sichtungskategorien für den Vergleich der Sichtungsalgorithmen herangezogen.

Für die vorliegende Studie wurden die klinischen Algorithmen *Manchester Triage System* (MTS, Modul MANV [22]), *Emergency Severity Index* (ESI [23, 30]), der Berliner Sichtungsalgorithmus (BER [24]), die prähospitalen Algorithmen *Primäres Ranking zur Initialen Orientierung im Rettungsdienst* (PRIOR [26]) und *Modified Simple Triage and Rapid Treatment* (mSTaRT [31]), sowie 2 Sichtungsalgorithmen aus einem internationalen Zivilschutzprojekt des BBK mit dem Haschemitischen Königreich Jordanien, „JorD“ (innerklinisch, Zusatzmaterial online: Abb. S1 [27]) und „PETRA“ (prähospital, Zusatzmaterial online: Abb. S2 [28]) verwendet. Ziel dieser internationalen Zusammenarbeit ist es, die zivile jordanische Gefahrenabwehr durch Ausstattungshilfe, Ausbildung und konzeptionelle Beratung zu stärken. Im Teilprojekt „Gesundheitlicher Bevölkerungsschutz“ werden prähospitale und klinische Aspekte betrachtet und ausgebildet. Der prähospitale Sichtungsalgorithmus PETRA wurde am BBK 2018 von jordanischen Paramedics des Jordan Civil Protection Departments sowie von deutschen Experten entwickelt, später mit der jordanischen Zivilschutzbehörde (Civil Defence Directorate) abgestimmt und seitdem landesweit ausgebildet. Für den innerklinischen Sichtungsalgorithmus arbeiteten jordanische und deutsche Ärzte zusammen. In dem Workshop wurden 2018 am BBK Kriterien erarbeitet, die später zu einem Algorithmus zusammengefügt und dem jordanischen Gesundheitsministerium zur weiteren Verwendung und Anpassung zur Verfügung gestellt wurden. Da die prähospitalen Algorithmen PRIOR und mSTaRT in Vorarbeiten bereits evaluiert worden waren [6] und in ihrer Anwendung in Deutschland verbreitet sind, wurden diese Verfahren zur externen Validierung des neuen Referenzdatensatzes mitgeführt.

Alle Algorithmen wurden in Microsoft Excel-Syntax (Microsoft, München, Deutschland) übersetzt, sodass für jeden Patientenfall in der Excel-Datenbank eine Sichtungskategorie entsprechend der jeweiligen Algorithmen automatisiert berechnet werden konnte (Zusatzmaterial online. Tab. S1–S7). Daneben gibt die Excel Syntax auch aus, nach wie vielen Schritten der jeweilige Algorithmus verlassen wurde. Hierzu musste die Datenbank in ein annähernd binäres Format umgewandelt werden, indem die Spalten das Ergebnis der jeweiligen Abfrage der Algorithmen enthielten. Um die Komplexität der Datenbank einzugrenzen, wurden sinngemäß ähnliche Abfragen der Algorithmen zusammengefasst (z. B. „instabiles Becken“ und „Beckenfraktur“ zur gemeinsamen Spalte „Beckenfraktur“, „Atemfrequenz > 29/min“ und „Atemfrequenz > 20/min“ zur gemeinsamen Spalte „Atemfrequenz > 29/min“, „FAST positiv“ und „FAST negativ“ zur gemeinsamen Spalte „FAST“ …). Eine Übersicht der Anpassungen und der Abfragen der Algorithmen ist im Zusatzmaterial online zu finden. Das Ergebnis der Abfragen der Algorithmen wurde wie folgt kodiert:

- „0“ → Abfrage ist mit „nein“ zu beantworten,
- „1“ → Abfrage ist mit „ja“ zu beantworten,
- „*n*“ → Ergebnis der Abfrage ist aus dem vorhandenen Datensatz inhaltlich nicht ableitbar.

Bei der Abfrage nach der vermuteten Anzahl an benötigten Ressourcen im Algorithmus ESI wurde entsprechend den ESI-Vorgaben „0 – keine Ressourcen“, „1 – eine Ressource“ und „2 – viele Ressourcen“ kodiert [23]. Die ESI-Level 1 und 2 wurden den Sichtungskategorien SK I (rot) bzw. SK II (gelb) zugeordnet. Die ESI-Level 3–5 wurden in der SK III (grün) zusammengefasst.

Nach entsprechender Vorbereitung der Datenbank wurden die Sichtungskategorien und die Anzahl der Algorithmusschritte bis zum Feststehen einer Entscheidung entsprechend den jeweiligen Algorithmen automatisiert für jede Patientenvignette berechnet. Anschließend wurden diese von den Algorithmen erzeugten Sichtungskategorien im Vergleich zur Referenzsichtungskategorie hinsichtlich ihrer diagnostischen Güte analog unserer prähospitalen Voruntersuchung [6] ausgewertet. Die statistische Auswertung erfolgte mit Microsoft Excel. Bestimmt wurden Sensitivität, Spezifität, NPV, PPV und Youden-Index für die Sichtungskategorien I–III. Der Youden-Index [32] fasst die Sensitivität und Spezifität gleichwertig zusammen (Youden-Index = Sensitivität + Spezifität − 1). Außerdem wurden die Algorithmen auch hinsichtlich einer Über- und Untertriage bewertet. Dabei ist zu berücksichtigen, dass eine Patientenvignette mit der SK I (rot) neben ihrer korrekten Einstufung nur untertriagiert werden kann (Tab. 1). Bei SK II (gelb) sind neben der korrekten Einstufung beide anderen Ausgänge möglich (Tab. 2). Bei der SK III (grün) kann nur korrekt oder übertriagiert werden (Tab. 3).

**Table 1:** 

Sichtungskategorie I (rot)		BER	ESI	MTS	JorD	PETRA	PRIOR	mSTaRT
*Sensitivität*		1,00	0,80	0,57	1,00	0,73	1,00	0,92
*Spezifität*		0,89	0,89	0,99	0,88	0,99	0,67	0,92
*PPV*		0,73	0,70	0,97	0,71	0,95	0,48	0,78
*NPV*		1,00	0,94	0,88	1,00	0,92	1,00	0,97
*Youden-Index*		0,89	0,69	0,57	0,88	0,72	0,67	0,84
*Untertriage*		0,0 %	20,4 %	42,9 %	0,0 %	26,5 %	0,0 %	8,2 %
*Algorithmusschritte*	Korrekt	3 (2–3)	1 (1–1)	4 (4–4)	1 (1–3)	6 (6–6)	3 (2–3)	3 (3–4)
	Untertriage	Keine	2 (2–2)	5 (1–5)	Keine	1 (1–8)	Keine	1 (1–3,5)
	Gesamt	3 (2–6)	1 (1–2)	4 (4–5)	1 (1–4)	6 (6–8)	3 (2–4)	3 (3–5)

Algorithmusschritte als Mediane (*IQR*), Signifikanzwerte im Zusatzmaterial online: Tab. S8

*PPV* positiv prädiktiver Vorhersagewert, *NPV* negativ prädiktiver Vorhersagewert. Der* Youden-Index* [32] fasst Sensitivität und Spezifität in einem Wert zusammen und steigt mit der Trennschärfe der Algorithmen, *BER* Berliner Sichtungsalgorithmus [24], *ESI* Emergency Severity Index [23], *MTS* Manchester Triage System – MANV Modul [22], *JorD* Jordanisch-Deutscher Projektalgorithmus Klinik [27], *PETRA* Prehospital Emergency Triage Rapid Algorithm [28], *PRIOR* Primäres Ranking zur Initialen Orientierung im Rettungsdienst [26], *mSTaRT* Modified Simple Triage and Rapid Treatment [16]

**Table 2:** 

Sichtungskategorie II (gelb)		BER	ESI	MTS	JorD	PETRA	PRIOR	mSTaRT
*Sensitivität*		0,38	0,22	0,16	0,11	0,27	0,02	0,13
*Spezifität*		0,90	0,78	0,73	0,99	0,75	0,99	0,81
*PPV*		0,52	0,21	0,14	0,83	0,22	0,33	0,16
*NPV*		0,84	0,79	0,76	0,80	0,79	0,79	0,77
*Youden-Index*		0,28	0,00	−0,11	0,11	0,01	0,01	−0,06
*Übertriage*		35,6 %	33,3 %	2,2 %	40,0 %	4,4 %	51,1 %	24,4 %
*Untertriage*		26,7 %	44,4 %	82,2 %	48,9 %	68,9 %	46,7 %	62,2 %
*Algorithmusschritte*	Korrekt	10 (9–14)	2 (2–2)	5 (5–5)	9 (9–9)	8 (8–8)	7 (7–7)	6 (6–6)
	Übertriagiert	3 (2–3)	1 (1–1)	4 (4–4)	4 (2–4)	6 (6–6)	5 (3–5)	5 (3–5)
	Untertriagiert	17 (17–17)	4 (4–4,5)	1 (1–1)	11 (11–11)	1 (1–1)	8 (8–8)	1 (1–1)
	Gesamt	9 (3–17)	2 (1–5)	1 (1–5)	9 (4–11)	1 (1–8)	6 (5–8)	1 (1–6)

Algorithmusschritte als Mediane (*IQR*), Signifikanzwerte im Zusatzmaterial online: Tabelle S8

*PPV* positiv prädiktiver Vorhersagewert, *NPV* negativ prädiktiver Vorhersagewert. Der* Youden-Index* [32] fasst Sensitivität und Spezifität in einem Wert zusammen und steigt mit der Trennschärfe der Algorithmen, *BER* Berliner Sichtungsalgorithmus [24], *ESI* Emergency Severity Index [23], *MTS* Manchester Triage System MANV Modul [22], *JorD* Jordanisch-Deutscher Projektalgorithmus Klinik [27], *PETRA* Prehospital Emergency Triage Rapid Algorithm [28], *PRIOR* Primäres Ranking zur Initialen Orientierung im Rettungsdienst [26], *mSTaRT* Modified Simple Triage and Rapid Treatment [16]

**Table 3:** 

Sichtungskategorie III (grün)		BER	ESI	MTS	JorD	PETRA	PRIOR	mSTaRT
*Sensitivität*		0,84	0,74	0,72	0,97	0,68	0,72	0,72
*Spezifität*		0,87	0,78	0,51	0,77	0,59	0,78	0,67
*PPV*		0,89	0,80	0,65	0,84	0,67	0,80	0,73
*NPV*		0,82	0,71	0,60	0,96	0,60	0,70	0,66
*Youden-Index*		0,72	0,52	0,23	0,74	0,27	0,50	0,39
*Übertriage*		15,5 %	25,9 %	27,6 %	2,6 %	31,9 %	27,6 %	28,4 %
*Algorithmusschritte*	Korrekt	17 (17–17)	5 (5–5)	1 (1–1)	11 (11–11)	1 (1–1)	8 (8–8)	1 (1–1)
	Übertriagiert	8 (8–10)	2 (2–2)	5 (5–5)	4 (2–9)	8 (8–8)	6 (6–6)	6 (6–6)
	Gesamt	17 (17–17)	5 (2–6)	1 (1–5)	11 (11–11)	1 (1–8)	8 (6–8)	1 (1–6)

Algorithmusschritte als Mediane (*IQR*), Signifikanzwerte im Zusatzmaterial online: Tab S8

*PPV* positiv prädiktiver Vorhersagewert, *NPV* negativ prädiktiver Vorhersagewert. Der* Youden-Index* [32] fasst Sensitivität und Spezifität in einem Wert zusammen und steigt mit der Trennschärfe der Algorithmen, *BER* Berliner Sichtungsalgorithmus [24], *ESI* Emergency Severity Index [23], *MTS* Manchester Triage System MANV Modul [22], *JorD* Jordanisch-Deutscher Projektalgorithmus Klinik [27], *PETRA* Prehospital Emergency Triage Rapid Algorithm [28], *PRIOR* Primäres Ranking zur Initialen Orientierung im Rettungsdienst [26], *mSTaRT* Modified Simple Triage and Rapid Treatment [16]

Die inferenzstatistische Auswertung der Algorithmusschritte erfolgte mit SPSS Version 24 (IBM, Ehningen, Deutschland). Bei wie hier vorliegend fehlender Varianzgleichheit sind Mediane mit Interquartilsabstand (IQR) angegeben. Zum statistischen Vergleich der Anzahl von Schritten bis zum Entscheid durch die Algorithmen wurde eine univariate Varianzanalyse mit dem Dunnett-T3-Post-hoc-Test für multiples Testen bei fehlender Varianzgleichheit eingesetzt. Eine statistische Signifikanz wurde bei *p* < 0,05 angenommen.

## Ergebnisse

Nach initialer Entfernung von Dubletten bzw. von Vignetten, zu denen im vorbereitenden Delphi-Prozess keine Einheitlichkeit in der Bewertung gefunden werden konnte, standen 210 Patientenvignetten für den Aufbau der Referenzdatenbank zur Verfügung. Die Rücklaufrate der Bewertung lag aufgrund der individuellen vertraglichen Vereinbarung des BBK mit allen Sichtenden bei 100 %. Alle Expertensichtungen konnten zur Auswertung herangezogen werden. Somit lagen für die Bildung der Patientenvignettenreferenzdatenbank 7560 Sichtungsvorgänge von 36 Sichtenden vor. Die aus dieser Referenzbildung hervorgegangenen medianen Sichtungsergebnisse für die 210 Patientenvignetten bildeten den Goldstandard für den Vergleich der 7 betrachteten Sichtungsalgorithmen miteinander.

**Figure 1:**
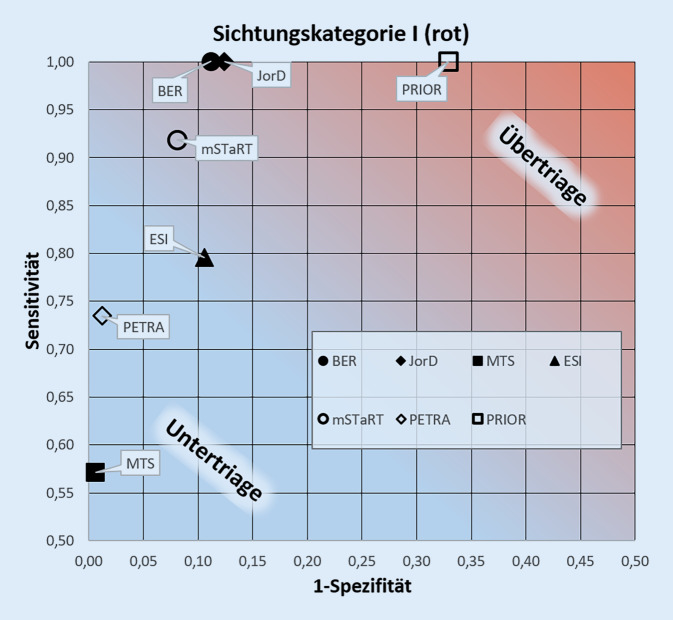

Abb. 1 zeigt die Auswertung der Algorithmen hinsichtlich der Sichtungskategorie I (rot) in einer Receiver-Operating-Charakteristik. Detaillierte Ergebnisse sind in Tab. 1 dargestellt. Die berechneten Sensitivitäten für die Detektion von Patienten der Sichtungskategorie I lagen zwischen 1,0 (Berliner Algorithmus, JorD und PRIOR) und 0,57 (MANV-Modul MTS). Die Spezifitäten lagen zwischen 0,99 (MTS und PETRA) und 0,67 (PRIOR). Die höchste Sensitivität zur Detektion einer Sichtungskategorie I (rot) erreichten der Berliner Sichtungsalgorithmus, JorD und PRIOR. Die höchste Spezifität zeigten die Algorithmen MTS und PETRA. Bei Betrachtung des Youden-Index ergab sich für den Berliner Sichtungsalgorithmus die beste Gesamtperformance (0,89), unmittelbar gefolgt vom innerklinischen Jordanisch-Deutschen Projektalgorithmus (*JorD*) mit 0,88. Von den hier ausgewerteten Algorithmen neigt am ehesten PRIOR zu einer Über- und das MANV-Modul von MTS zu einer Untertriage.

Insgesamt ist die Genauigkeit der Algorithmen für die Detektion von Patienten der Sichtungskategorien II (gelb) und SK III (grün) deutlich schlechter als für die der Sichtungskategorie I (rot). Für die Sichtungskategorie II (gelb) (Tab. 2) lagen die berechneten Sensitivitäten zwischen 0,38 (BER) und 0,02 (PRIOR). Die Spezifitäten lagen zwischen 0,99 (JorD und PRIOR) und 0,73 (MTS). Bei Betrachtung des Youden-Index zeigte ebenfalls der Berliner Sichtungsalgorithmus die beste Gesamtperformance (0,28).

Für die Detektion von Patienten der Sichtungskategorie III (grün) (Tab. 3) lagen die berechneten Sensitivitäten zwischen 0,97 (JOR) und 0,68 (PETRA). Die Spezifitäten lagen zwischen 0,87 (BER) und 0,51 (MTS). Bei Betrachtung des Youden-Index zeigten JorD (0,74) und BER (0,72) für die Erkennung von Patienten der Sichtungskategorie III (grün) die beste Gesamtperformance.

Über alle Sichtungskategorien hinweg zeigten sich bis zum Feststehen einer Entscheidung durch die unterschiedlichen Algorithmen folgende Schrittanzahlen (Median [IQR]) in absteigender Reihenfolge: BER 17 [3–17]; JorD 11 [4–11]; PRIOR 7,5 [4–8]; ESI 4 [1–5]; PETRA 1 [1–8] und 1 [1–4] Schritte, sowohl mit mSTaRT als auch dem MANV Modul von MTS. Die Tab. 1, 2 und 3 geben die jeweiligen Schrittanzahlen, getrennt nach Sichtungskategorien und erzielter Korrektheit, an. Die Unterschiede wiesen in der ANOVA einen *p*-Wert < 0,001 auf. Die Ergebnisse der Einzelvergleiche sind im Zusatzmaterial online: Tab S8 angegeben.

## Diskussion

In den letzten Jahren wurden vermehrt Studien durchgeführt, um prähospitale Vorsichtungsalgorithmen zu evaluieren [6, 13–17]. Demgegenüber fehlen Untersuchungen zu klinischen Sichtungsalgorithmen. Insbesondere für den gegenwärtigen Leitlinienprozess in der innerklinischen Katastrophenmedizin [33] sind belastbare Empfehlungsgrundlagen nicht verfügbar. Aus diesem Grund hatte die vorliegende Studie das Ziel, bereits existierende und neu entwickelte klinische Sichtungsalgorithmen zu evaluieren und zu vergleichen. Eines der Hauptergebnisse dieser Studie ist die erstmalige Etablierung eines Masterdatensatzes von 210 Verletztenvignetten, die von insgesamt 36 nationalen Experten validiert wurden. Dieser Masterdatensatz bietet für die Zukunft die Möglichkeit, neue Sichtungsalgorithmen zu validieren und existierende Algorithmen zu verbessern. Um eine Vergleichbarkeit mit Studien zu prähospitalen Vorsichtungsalgorithmen herzustellen, wurden in die Berechnungen auch PRIOR [26], mSTaRT [16] und PETRA (Zusatzmaterial online: Abb. S2) integriert.

Vor‑/Sichtungsalgorithmen haben zum Ziel, Patienten eines Massenanfalls von Verletzten (MANV), entsprechend ihrem Verletzungsmuster, möglichst präzise in eine Sichtungskategorie einzustufen. Hierbei besitzt die Sichtungskategorie I (rot) eine besondere Relevanz, da für diese Patienten unmittelbare und akute Lebensgefahr besteht [8, 9]. Daher ist es von großer Bedeutung, dass Vor‑/Sichtungskonzepte insbesondere diese Patienten zuverlässig erkennen. In vorgegangenen Studien [6, 14, 15] konnten wir zeigen, dass insbesondere die Algorithmen der START-Familie diesen Anspruch prähospital erfüllen, während PRIOR [26] maßgeblich über- bzw. der Field Triage Score [34] erheblich untertriagiert. In der vorliegenden Untersuchung ergab sich für die Patienten der Sichtungskategorie I (rot) über alle Verfahren hinweg eine Sensitivität von überwiegend ≥ 0,8 (Tab. 1). Als negativer Ausreißer fällt hierbei das MANV-Modul der Manchester Triage (MTS) mit einer Sensitivität von lediglich 0,57 auf. Ebenso konnte für die meisten Algorithmen eine hohe Spezifität für die Detektion der Sichtungskategorie I (rot) von deutlich über 0,8 gezeigt werden (Tab. 1). Als Schlusslicht zeigte sich hier ähnlich wie in der prähospitalen Evaluation [6] die Spezifität des PRIOR-Algorithmus mit 0,67.

Damit lässt sich festhalten, dass die untersuchten Algorithmen dem Anspruch einer präzisen Erkennung der Patienten der SK I in unterschiedlichem Maß gerecht werden. Dies deckt sich ebenso mit vorangegangenen Studien [6, 16, 35]. Allerdings ist es ein immanentes Problem aller diagnostischen Tests, dass sich eine hohe Sensitivität, also hier die Erkennung aller lebensbedrohlich Verletzten, nur zulasten der Spezifität erreichen lässt und eine Übertriagierung wahrscheinlicher macht (Abb. 1). Um eine balancierte Betrachtung von Sensitivität und Spezifität (hier Gefahr Untertriagierung) zu ermöglichen, wurde der Youden-Index [32], der sowohl Sensitivität als auch Spezifität zu einem Rechenwert zusammenfasst und eine höhere Diskriminierungsfähigkeit mit steigenden Werten anzeigt, für alle untersuchten Algorithmen angegeben.

Hinsichtlich der Vergleichbarkeit von prähospitalen zu klinischen Algorithmen zeigt sich eine weitgehend gute Übereinstimmung (Abb. 1). Dies ist insbesondere aus prozessqualitativer Sicht positiv hervorzuheben, da dadurch der Transfer von Informationen aus der Präklinik in die Klinik erleichtert wird [5]. Insbesondere korrespondieren die prähospitalen Vorsichtungsergebnisse bei Zugrundelegung der gewählten Zuordnung mit den ESI-Levels. Dies ermöglicht es den jeweiligen Teams (prähospital vs. klinisch), ihre gewohnten Algorithmen anzuwenden, ohne dass dadurch Brüche in der Einstufung in die Sichtungskategorien resp. der ESI-Level entstehen. Bemerkenswert einheitliche Ergebnisse haben die Berechnungen insbesondere für die Übertragung von SK I auf ESI-Level 1 ergeben. Außerdem zeigen die Daten, dass die Patienten der SK III grundsätzlich in den ESI-Levels 3–5 zusammengefasst werden müssen. Eine genauere Unterteilung der ESI-Levels 3–5 kann dann in den Notaufnahmen z. B. in den Eingangssichtungen erfolgen.

Die höchste Testgüte für die Detektion der SK I (rot) lieferte jedoch der Berliner Sichtungsalgorithmus. Mit einer Sensitivität von 1,0 und einer Spezifität von 0,89 ist er, bezogen auf Über- und Untertriage, der ausgewogenste Algorithmus dieser Evaluation; allerdings dicht gefolgt vom innerklinischen Jordanisch-Deutschen Projektalgorithmus (JorD) für die klinische Sichtung (Zusatzmaterial online: Abb. S1, [27]), und von mSTaRT [16]. Die in Notaufnahmen eingesetzten MTS- und ESI-Algorithmen zeigten im Vergleich schlechtere Ergebnisse, weshalb die Autoren die Vorhaltung einen speziellen klinischen Sichtungsalgorithmus für MANV empfehlen.

Das Akaike-Informationskriterium [36] fordert, dass ein Modell das eine geringere Komplexität aufweist, bei gleicher Güte zu bevorzugen ist. Die Anzahl der Parameter bzw. Abfrage-Items wird dabei „strafend“ berücksichtigt. Übertragen auf Sichtungsalgorithmen ist ein einfacherer Algorithmus schneller in der Durchführung [6, 37] und auch leichter zu schulen [18, 38]. Zudem können einfache Algorithmen auch unproblematisch in Form von schneller abzuarbeitenden Checklisten ausgeführt werden [39, 40], allerdings ggf. mit der Einschränkung einer geringeren Präzision, wie später weiter ausgeführt wird.

In der vorliegenden Arbeit konnte die Zeitdauer der Algorithmendurchläufe anhand der computergestützten Simulation nicht vergleichend bestimmt werden, wie es mit menschlichen Sichtern möglich gewesen wäre. Hilfsweise kann nur die Anzahl der durchlaufenen Schritte bis zum Ergebnis herangezogen werden [6]. Aus einer Evaluierungsstudie des PRIOR-Algorithmus [37] ist bekannt, dass die die Sichtung der SK-III-Patienten mit 42 s sowohl gegenüber den anderen SK als auch dem mSTaRT-Algorithmus am längsten gedauert hat. Der Sichtungsvorgang wurde bei PRIOR für SK I, II, III mit 27, 28 resp. 42 s und bei mSTaRT mit 35, 20 resp. 10 s angegeben. Werden Zeitansätze verglichen, muss die Verteilung der SK in der betrachteten Kohorte miteinfließen. Bei einer Patientenverteilung SK I, II, III, EX von 15 %, 20 %, 60 % resp. 5 % in 100 Patienten, die nach PRIOR gesichtet werden, entstehen Zeitaufwände für die Sichtung der SK III von 42 min. Im Vergleich dazu liegt der Zeitaufwand bei mSTaRT für diese Kategorie bei 10 min. Verschiebt sich die Patientenverteilung noch weiter zugunsten der SKIII wie bei der von Brüne [2] gefundenen Gesamtverteilung bei MANV (SK I, II, III von 7 %, 19 % resp. 74 %), dann werden mit dem PRIOR-Verfahren 81 % der Sichtungszeit bei Leichtverletzten verwendet. Variationen der Item-Abfolge innerhalb eines Algorithmus [15] oder ihr vorzeitiger Abbruch können sowohl die diagnostische Genauigkeit als auch die Dauer bis zum Entscheid je nach Algorithmus positiv oder negativ beeinflussen.

Über die Sichtungskategorien hinweg benötigen sowohl BER mit 17 [3–17] als auch JorD mit 11 [4–11] signifikant mehr Schritte als alle anderen Algorithmen. Doch diese isolierte und übergreifende Betrachtung eignet sich nicht, um den Erfüllungsgrad der Aufgabenstellung von Sichtungsalgorithmen zu beurteilen. Nach der Vorgabe der 7. Sichtungskonsensuskonferenz [18] sollen Algorithmen folgende Eigenschaften in absteigender Gewichtung besitzen:

- Patienten der SK I (rot) schnell identifizieren,
- zuverlässige Identifizierung (Über- und Untertriage vermeiden),
- geringer Zeitaufwand,
- einfach anwendbar,
- einfach erlernbar.

**Figure 2:**
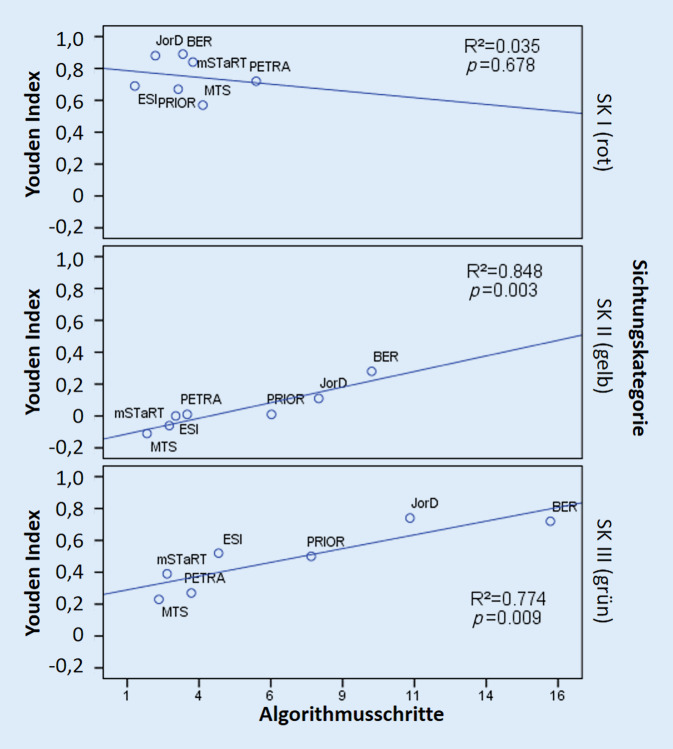

Daher ist eine vergleichende Betrachtung des Zeitaufwandes oder die eines Surrogatparameters wie der Anzahl von Algorithmusschritten *nach Sichtungskategorien* maßgeblich. Die wenigsten Schritte bis zur korrekten Identifikation der SK-I(Rot)-Patienten benötigen ESI (1 [1–1]) und JorD (1 [1–3]), ESI triagiert dabei aber 20,4 % der tatsächlich SK-I(Rot)-Patienten in die SK II (gelb) (Tab. 1). Allerdings findet sich entsprechend Abb. 2 oben kein statistischer Zusammenhang zwischen Testgüte und den Algorithmusschritten bei der SK I (rot). Die Problematik einfacherer Sichtungsalgorithmen spiegelt sich gerade in der Diskrimination von SK-II(Gelb)-Patienten wider, da hier eine Fehleinschätzung in beide Richtungen möglich ist [24]. Dieser Effekt wurde im Rahmen der Entwicklung und Validierung des Berliner Sichtungsalgorithmus nachgewiesen und zugunsten einer besseren Diskrimination von 5 gelben Diskriminanten auf 9 erhöht [24]. Entsprechend werden beim BER für eine korrekte Identifizierung von Patienten der SK II mit insgesamt 10 [9–14] die meisten Schritte benötigt (Tab. 2). Dadurch wurde eine Verbesserung der Treffsicherheit, allerdings zuungunsten der Anzahl der Diskriminanten erzielt. Eine höhere Treffsicherheit für die SK II hat, wie auch Abb. 2*Mitte* zeigt, den Preis eines komplexeren Algorithmus (SK II, Youden-Index BER 0,28 vs. JorD 0,11).

Betrachtet man den mutmaßlichen Zeitaufwand für die Identifikation von Leichtverletzten (SK III (grün)), so muss konstatiert werden, dass auch hier die Algorithmen mit der besten Diskriminierungsfähigkeit die meisten Schritte und damit annehmbar auch die längste Zeit für eine korrekte Entscheidung benötigen Abb. 2 unten: BER (17 [17–17]) vs. JorD (11 [11–11]) Schritte. Dies ist jedoch durch die Priorisierung der Detektion von SK-I-Patienten bedingt. Bei regulär funktionierendem Rettungsdienst mit Transportpriorisierung werden in Kliniken auch zuerst SK-I-Patienten eintreffen. Ausnahme stellen hier Szenarien mit relevanter Anzahl an Selbsteinweisern dar. Weiter oben wurde dieses Problem bereits für den PRIOR-Algorithmus diskutiert: Wenn bei einem zufälligen Eintreffen klar ist, dass der größte Zeitaufwand für die Identifikation der Leichtverletzten besteht, dann muss sichergestellt werden, dass kein Schwerverletzter in einer Warteschlange die korrekte Identifikation von Leichtverletzten abwarten muss. Zur Lösung dieses Dilemmas wurde bereits in der 8. Sichtungskonsensuskonferenz eine Zugangskoordination zum Sichtungspunkt gefordert. „*Lageabhängig kann ärztliches oder medizinisches Fachpersonal den Zugang zur Klinik koordinieren mit dem Ziel, offensichtlich vital bedrohte Patienten zu identifizieren. Dadurch sollen diese Patienten schneller dem Sichtungsprozess zugeführt werden. Dieses Vorgehen ersetzt nicht die klinische Sichtung*“ [8].

Unabhängig davon konnte für den prähospitalen PRIOR-Algorithmus gezeigt werden, dass die schlichte Umstellung des Abfrage-Items „Gehfähigkeit“ vom Ende an den Anfang des Algorithmus nicht nur dessen Diskriminierungsfähigkeit deutlich verbessert [15]: Es ergibt sich weiterhin, dass sich die Gesamtzahl der für eine Patientenkohorte abzuarbeitenden Algorithmusschritte durch eine solche Umstellung abnimmt. Entsprechend wäre zu prüfen, ob die hier untersuchten Algorithmen durch eine ähnliche Umstellung der Abfrage-Items noch weiter verbessert werden können.

Einen weiteren Aspekt stellt der zeitliche Aufwand der administrativen Aufnahme in den Kliniken dar. Aus Erfahrung der Autoren benötigt dies am Sichtungsplatz trotz vorbereiteter Katastrophenakten am meisten Zeit, sodass der zeitliche Aufwand des Sichtungsalgorithmus selbst eher in den Hintergrund tritt.

Unter Berücksichtigung der geführten Diskussion muss letztlich der Anwender entscheiden, welche Strategie er im Rahmen der klinischen Sichtung verfolgt. Hierbei ist v. a. der Zeitaufwand eines komplexeren Algorithmus am Sichtungsplatz gegen die möglicherweise schlechtere diagnostische Güte eines einfacheren und schnelleren Algorithmus abzuwägen. Bei weniger ausgeprägtem Ressourcenmangel wirkt sich die Übertriagierung eines Sichtungsalgorithmus weniger auf die Konkurrenz der wahren SK-I-Patienten (richtig Positive) um die medizinischen Ressourcen mit den falsch zugeordneten SK-I-Patienten (falsch Positive) aus. Aus Sicht der Algorithmenausbildung, der permanenten Wissensverfügbarkeit und Anwendungssicherheit ist allerdings davon abzuraten, lage-/ressourcenentsprechend unterschiedliche Algorithmen vorhalten zu wollen [8].

Ein weiterer Diskussionspunkt ist der Einsatz einer fokussierten Ultraschalluntersuchung (FAST) [41] in Algorithmen bzw. der Eingangssichtung. Grundsätzlich erscheint es sinnvoll, innerklinisch vorhandene Ressourcen, die zur besseren Diskriminierung des Patientenzustands beitragen können, in den Sichtungsprozess einzubeziehen, die prähospital nicht realistisch verfügbar und einsetzbar sind. So kann Personal, dass in FAST geschult und erfahren ist, grundsätzlich wertvolle Informationen liefern, die die nächsten Behandlungsschritte beeinflussen. Gleichzeitig ist zu berücksichtigen, dass eine Konzentration des FAST auf diejenigen Patienten, die einem sensitiven Vorselektionsverfahren wie der Sichtung anhand klinischer Parameter unterzogen worden sind, die Spezialistenressource zielgenauer einsetzt. Werden szenarioabhängige Kategorieverteilungen von SK I (20 %), SK II (30 %) und SK III (50 %) zugrunde gelegt [2, 9, 18, 42], dann sind mehr als 50 % der FAST-Untersuchungen verzichtbar, zugunsten der Möglichkeit, sich ausführlicher mit den Patienten der SK I im Behandlungsbereich rot zu befassen [3, 41]. Im Ausbildungskonzept des Berliner Sichtungsalgorithmus ist dies so berücksichtigt. Nur Patienten mit stumpfen Bauchtrauma ohne akut vitale Bedrohung erhalten am Sichtungspunkt eine FAST, um hier Patienten mit freier intraabdomineller Flüssigkeit (SK I) von Patienten ohne (SK II) unterscheiden zu können. Solches Personal ist in Notaufnahmen regelhaft vorhanden. In der Präklinik sind die Schulung und der Einsatz des FAST hingegen noch weniger etabliert [43].

Das BBK hat die Entwicklung des zugrunde liegenden Patientendatensatzes mitfinanziert, der zukünftig für nationale und internationale Ausbildungen und Übungen in der Krankenhausalarm- und -einsatzplanung genutzt werden kann. Das Evaluationsergebnis der im internationalen Zivilschutzprojekt des BBK mit dem Haschemitischen Königreich Jordanien entwickelten Sichtungsalgorithmen, ist eine Bestätigung für die gelungene internationale Projektzusammenarbeit und erhöht die Akzeptanz der Algorithmen bei Entscheidungsträgern in beiden Partnerländern.

Limitierend ist in der vorliegenden Analyse festzustellen, dass die weiterentwickelten Patientenvignetten aus den Berliner Krankenhauseinsatzübungen [25] anders als in unserer prähospitalen Vorstudie aus realen Patientenfällen [6] alle fiktive Fallbeispiele darstellen. Außerdem ist ein limitierender Faktor unserer Studie das Design der Datenbank selbst. Da die Abfragen der Vor‑/Sichtungsalgorithmen z. T. sehr konkret und speziell sind, existierte nicht zu jedem Algorithmus-Item 1 zu 1 eine Spalte mit der jeweiligen Information des Patientenbeispiels und musste komplex nachberechnet werden. In einer nachfolgenden Studie sollte daher stärker darauf geachtet werden, dass alle Abfragen der zu untersuchenden Algorithmen direkt in der Patientendatenbank widergespiegelt sind.

## Schlussfolgerung

In der vorliegenden Studie konnte eine Übertragbarkeit prähospitaler algorithmenbasierter Vorsichtungsergebnisse auf die Ergebnisse der klinischen Algorithmen gezeigt werden. Die höchste diagnostische Güte für die innerklinische Sichtung lieferte der Berliner Sichtungsalgorithmus, gefolgt vom innerklinischen Jordanisch-Deutschen Projektalgorithmus (JorD), die allerdings auch die meisten Algorithmusschritte bis zum Entscheid benötigen. Für die Erkennung der Sichtungskategorien SK II und SK III besteht ein Zusammenhang zwischen der Anzahl von Algorithmusschritten und der erreichten Testgüte.

In einer nachfolgenden Studie müssen die Ergebnisse an realen Patientendatensätzen z. B. aus Notaufnahmen validiert werden. Weiterer Forschungsbedarf ergibt sich weiterhin für eine mögliche Verbesserung der Algorithmen selbst.

## Supplementary Information




