# Supplementary material for: Validation of secondary triage algorithms for mass casualty incidents—A simulation-based study—German version
Source: Anaesthesiologie. 2023 Jun 15;72(7):467–76. [Article in German] doi: 10.1007/s00101-023-01291-3 (PMC10322965; doi:10.1007/s00101-023-01291-3)
Supplement: Supplementary file 1 [file 101_2023_1291_MOESM1_ESM.docx]

**Zusatzmaterial zum Beitrag** „**Validierung innerklinischer Sichtungsalgorithmen für den Massenfall von Verletzten- Eine simulationsbasierte Studie**“ von Heller AR, Neidel T, Klotz PJ et al. (2023) in *Die Anaesthesiologie*

Beitrag und Zusatzmaterial stehen Ihnen auf www.springermedizin.de zur Verfügung. Bitte geben Sie dort den Beitragstitel in die Suche ein.

#

# **Abbildung S1: Jordanisch- Deutscher Sichtungsalgorithmus für die Klinik (JorD) (**[**27**](#_ENREF_27)**)**


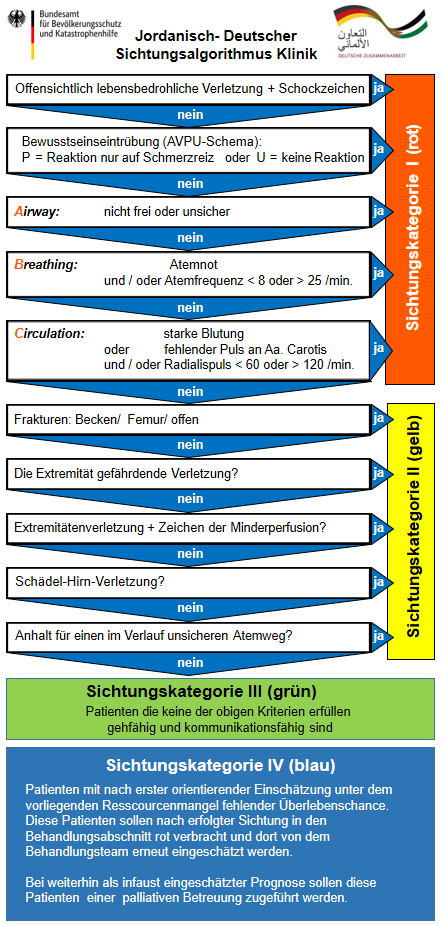


# **Abbildung S2: Jordanisch- Deutscher Sichtungsalgorithmus „Prehospital Emergency Triage Rapid Algorithm (PETRA) für die Präklinik (**[**28**](#_ENREF_28)**)**


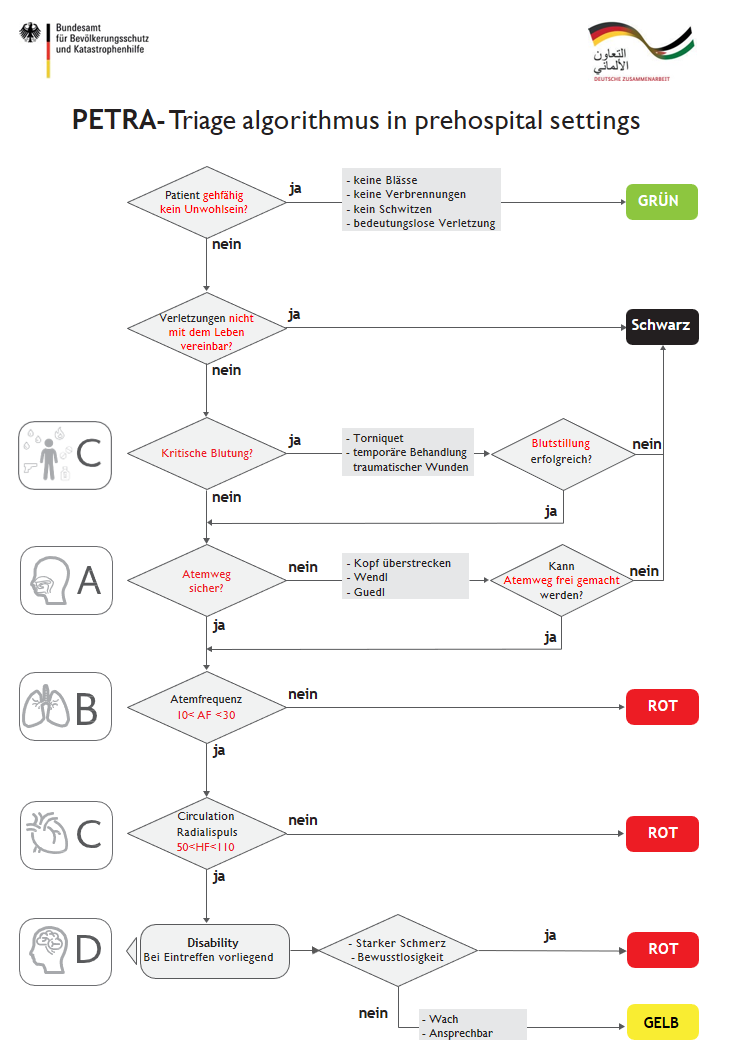


# **Computersimulation der Algorithmen**

Die für jeden Algorithmus geschachtelten WENN- Abfragen der Parameter aus der jeweils zugehörigen Parametertabelle ermitteln die entsprechende Sichtungskategorie und die Anzahl der bei jedem Patienten durchgeführten Algorithmusschritte. Die „WENN“-Funktion wird allgemein verwendet, um bedingte Tests für Werte und Formeln auszuführen und gibt einen Wert zurück, der davon abhängt, ob eine angegebene Bedingung als WAHR oder FALSCH ausgewertet wird.

Syntax: WENN(Prüfung;Dann_Wert;Sonst_Wert)

# **Tabelle S1: Parameterliste zur Abfrage durch die Excel- Syntax für den Berliner Algorithmus (BER) (**[**24**](#_ENREF_24)**)**

| **Abfrage** | **Spalte** |
| --- | --- |
| Fehlende Spontanatmung | T |
| GCS<9 | AN |
| Respiratorische Insuffizienz | W |
| Penetrierende Thoraxverletzung | AP |
| Atemnot | AA |
| Spritzende Blutung | AC |
| Mechanisch instabiles Becken | BH |
| Bauchtrauma | AU (stumpf) |
| Penetrierendes Bauchtrauma | AQ |
| FAST positiv | AD |
| Schock | AK |
| Rekapzeit > 2 s oder syst. RR <90 | AF oder AE |
| Inhalationstrauma | Z |
| Brustschmerz | AR |
| Atemnot | AA |
| Schwere Kopfverletzung | AM |
| Querschnitt/Lähmung | AL |
| Beckenfraktur | BH |
| Femurfraktur | AS |
| Offene Fraktur | AT |
| Bauchtrauma stumpf und FAST negativ | AU und AD negativ |
| Ausgedehnte Weichteilverletzung/Makroamputation | AV |
| Verbrennung > 20% KOF | AW |
| Stromunfall | AX |
| Penetrierende Augenverletzung | AY |
| SK III (grün) |  |

MS Excel-Syntax zur Ermittlung der Sichtungskategorien Berliner Algorithmus (BER) (21) mit Variablenbezug zur Tabelle S1:

=WENN(T2=0;"SK1(1)";WENN(AN2=1;"SK1(2)";WENN(ODER(W2=1;AP2=1;AA2=1);"SK1(3)";WENN(AC2=1;"SK1(4)";WENN(BH2=1;"SK1(5)";WENN(ODER(AU2=1;AQ2=1;AD2=1);"SK1(6)";WENN(ODER(AK2=1;AF2=1;AE2=1);"SK1(7)";WENN(Z2=1;"SK2(8)";WENN(ODER(AR2=1;AA2=1);"SK2(9)";WENN(AM2=1;"SK2(10)";WENN(AL2=1;"SK2(11)";WENN(ODER(BH2=1;AS2=1;AT2=1);"SK2(12)";WENN(UND(AU2=1;AD2=0);"SK2(13)";WENN(AV2=1;"SK2(14)";WENN(ODER(AW2=1;AX2=1);"SK2(15)";WENN((AY2=1);"SK2(16)";"SK3(17)"))))))))))))))))

# **Tabelle S2: Parameterliste zur Abfrage durch die Excel- Syntax für den Emergency severity Index (ESI) (**[**23**](#_ENREF_23)**)**

| **Abfrage** | **Spalte** |
| --- | --- |
| A: |  |
| Intubiert | U |
| Apnoe | T |
| Kein Puls | AG |
| Atemnot | AA |
| SpO2 <90 | W |
| Akute Bewusstseinsveränderung | AN |
| Nicht ansprechbar | AO |
|  |  |
| B |  |
| Thoraxschmerzen | AR |
| Kreislaufstörung | AU |
| Verwirrtheit | AN |
| Lethargie | AN |
| Desorientiertheit | AN |
| Starke Schmerzen | AZ |
|  |  |
| C |  |
| Benötigte Ressourcen | BJ |
| Keine -> 5 , eine -> 4 , viele -> D |  |
| D |  |
| HF>100 | AH |
| AF>20 | Y oder AA? |
| SpO2 <92 | W oder O |

MS Excel-Syntax zur Ermittlung der Sichtungskategorien für den Emergency severity Index (ESI) ([23](#_ENREF_23)) mit Variablenbezug zu Tabelle S2:

=WENN(ODER(U2=1;T2=0;AG2=0;AA2=1;W2=1;AN2=1;AO2=0);“EL1“;WENN(ODER(AR2=1;AU2=1;AN2=1;AZ2=1);“EL2“;WENN(UND(BJ2=2;(ODER(AH2=1;Y2=1;W2=1)));“EL2“;WENN(UND(BJ2=2;AH2=0;Y2=0;W2=0);“EL3“;WENN(BJ2=1;“EL4“;WENN(BJ2=0;“EL5“;“FEHLER“))))))

Fehlerausgabe wegen zu wenigen Angaben der AF in der Datenbank -> Umwandlung Abfrage AF in Abfrage Atemnot -> dann Ergebnis möglich

=WENN(ODER(U2=1;T2=0;AG2=0;AA2=1;W2=1;AN2=1;AO2=0);“EL1“;WENN(ODER(AR2=1;AU2=1;AN2=1;AZ2=1);“EL2“;WENN(UND(BJ2=2;(ODER(AH2=1;AA2=1;W2=1)));“EL2“;WENN(UND(BJ2=2;AH2=0;AA2=0;W2=0);“EL3“;WENN(BJ2=1;“EL4“;WENN(BJ2=0;“EL5“;“FEHLER“))))))

# **Tabelle S3: Parameterliste zur Abfrage durch die Excel- Syntax für das MANV Modul des Manchester Triage Systems (MTS) (**[**22**](#_ENREF_22)**)**

| **Abfrage** | **Spalte** |
| --- | --- |
| Gehfähig | BA |
| Spontanatmung | T und U (nicht intubiert) |
| AF <10 | X |
| AF > 29 | Y |
| Rekapzeit > 2s | AF |
| Puls über 120 | AH |
| SK II (gelb) |  |

MS Excel-Syntax zur Ermittlung der Sichtungskategorien für das MANV Modul des Manchester Triage Systems (MTS) ([22](#_ENREF_22)) mit Variablenbezug zu Tabelle S3:

=WENN(BA2=1;“SK3(1)“;WENN(UND(T2=0;U2=0);“EX(2)“;WENN(ODER(X2=1;Y2=1);“SK1(3)“;WENN(ODER(AF2=1;AH2=1);“SK1(4)“;“SK2(5)“))))

# **Tabelle S4: Parameterliste zur Abfrage durch die Excel- Syntax für den Jordanisch- Deutschen Projekt- Algorithmus für die Klinik ((JorD) SDC Abbildung 3 (**[**27**](#_ENREF_27)**))**

| **Abfrage** | **Spalte** |
| --- | --- |
| Lebensbedrohliche Verletzung + Schock | AK und BG |
| Bewusstseinseintrübung | AN |
| Atemweg nicht frei oder unsicher | T oder V + nicht intubiert |
| Atemnot | AA |
| AF <8 oder > 25 | X und Y |
| Starke Blutung | AC |
| Puls <60 oder >120 | AH und AI |
| Fehlender Puls | AG |
| Beckenfraktur | BH |
| Femurfraktur | AS |
| Offene Fraktur | AT |
| Extremität gefährdet | BI |
| Extremitätenverletzung + Zeichen der Minderperfusion | BI |
| Schädel-Hirn-Verletzung | AM |
| Unsicherer Atemweg | V |
| SK III (grün) |  |

MS Excel-Syntax zur Ermittlung der Sichtungskategorien für ((JorD) SDC Abbildung 3 (23)) mit Variablenbezug zu S4:

=WENN(UND(AK2=1;BG2=1);“SK1(1)“;WENN(AN2=1;“SK1(2)“;WENN(ODER(UND(T2=0;U2=0);V2=1);“SK1(3)“;WENN(ODER(AA2=1;X2=1;Y2=1);“SK1(4)“;WENN(ODER(AC2=1;AH2=1;AI2=1;AG2=0);“SK1(5)“;WENN(ODER(BH2=1;AS2=1;AT2=1);“SK2(6)“;WENN(BI2=1;“SK2(7/8)“;WENN(AM2=1;“SK2(9)“;WENN(V2=1;“SK2(10)“;“SK3(11)“)))))))))

# **Tabelle S5: Parameterliste zur Abfrage durch die Excel- Syntax für den Jordanisch- Deutschen Projekt- Algorithmus für die Präklinik ((PETRA) SDC Abbildung 4 (**[**28**](#_ENREF_28)**))**

| Abfrage | Spalte |
| --- | --- |
| Gehfähig + nicht blass + nicht verbrannt + kein Schweiß | BA + BC + BD + BE |
| Tödliche Verletzung | BB |
| Kritische Blutung | AC |
| Atemweg Frei | T und nicht intubiert |
| AF unter 10 oder über 30 | X und Y |
| Puls unter 50 oder über 110 | AI und AH |
| Disability |  |
| Bewusstlos | AO |
| Nicht ansprechbar | AO |
| SK II (gelb) |  |

MS Excel-Syntax zur Ermittlung der Sichtungskategorien für *PETRA (SDC Abbildung 4 (*[28](#_ENREF_28)*))* mit Variablenbezug zu *Tabelle* S5. Abfragen von „Tödliche Verletzung“ bis „AF unter….“ Nicht berücksichtigt, da ohne Einfluss auf die Sichtungskategorie.

=WENN(UND(BA2=1;BC2=0;BD2=0;BE2=0);“SK3(1)“;WENN(BB2=1;“EX(2)“;WENN(ODER(X2=1;Y2=1);“SK1(5)“;WENN(ODER(AI2=1;AH2=1);“SK1(6)“;WENN(AO2=0;“SK1(7)“;“SK2(8)“)))))

# **Tabelle S6: Parameterliste zur Abfrage durch die Excel- Syntax für PRIOR (**[**26**](#_ENREF_26)**)**

| Abfrage | Spalte |
| --- | --- |
| Lebensbedrohende Blutung | AC |
| Bewusstlos | AO |
| Unsicherer Atemweg | V |
| Atemstörung | AA und AB |
| Atemstillstand | T |
| AF deutlich gestört | Y und X |
| Kreislaufstörung | AJ und AK (Schock) |
| Kein Radialispuls | AE oder AG |
| Rekapzeit > 2 s | AF |
| Starke Blutung | AC |
| Bewusstseinsstörung | AN |
| Desorientiert, somnolent | AN |
| Starke schmerzen | AZ |
| Liegend | BA |

MS Excel-Syntax zur Ermittlung der Sichtungskategorien für *PRIOR (*[26](#_ENREF_26)*) mit Variablenbezug zu Tabelle S6*

=WENN(AC2=1;“SK1(1)“;WENN(ODER(AO2=0;V2=1);“SK1(2)“;WENN(ODER(T2=0;AA2=1;AB2=1;Y2=1;X2=1);“SK1(3)“;WENN(ODER(AJ2=1;AK2=1;AE2=1;AF2=1;AC2=1);“SK1(4)“;WENN(AN2=1;“SK1(5)“;WENN(AZ2=1;“SK1(6)“;WENN(BA2=0;“SK2(7)“;“SK3(8)“)))))))

# **Tabelle S7: Parameterliste zur Abfrage durch die Excel- Syntax für mSTaRT (**[**16**](#_ENREF_16)**)**

| Abfrage | Spalte |
| --- | --- |
| gehend | A |
| tödliche Verletzung | C |
| Atemstörung | D |
| Atemfrequenz | G |
| Radialispuls tastbar | H |
| spritzende Blutung | K |
| Anweisungen befolgen | L |

MS Excel-Syntax zur Ermittlung der Sichtungskategorien für *mSTaRT (*[16](#_ENREF_16)*) mit Variablenbezug zu Tabelle S7*

=WENN(A=1;"SK3(1)";WENN(C=1;"SK4(2)";WENN(D=1;"SK1(3)";WENN(G>30;"SK1(4)";WENN(G<10;"SK1(4)";WENN(K=1;"SK1(5)";WENN(H=0;"SK1(6)";WENN(L=0;"SK1(7)";"SK2(8)"))))))))

**Tabelle S8: Liste der Signifikanzniveaus für den Vergleich der Anzahl der Algorithmus- Schritte zwischen den unterschiedlichen Verfahren*.*** ANOVA mit post- hoc Dunnet T3- Korrektur bei Varianzungleichheit: Weiß Vergleich unabhängig von der Sichtungskategorie und farbig markiert entsprechend der Sichtungskategorien
